# Learning on mobile augmented reality trails of integrity and ethics

**DOI:** 10.1186/s41039-018-0088-6

**Published:** 2018-12-17

**Authors:** Eva Y. W. Wong, Theresa Kwong, Mark Pegrum

**Affiliations:** 1 Centre for Holistic Teaching and Learning, Hong Kong Baptist University, Kowloon Tong, Kowloon, Hong Kong; 2 Graduate School of Education, The University of Western Australia, Perth, Australia

**Keywords:** Improving classroom teaching, Interactive learning environments, Pedagogical issues, Postsecondary education

## Abstract

At the heart of university education, there must be an emphasis on students developing academic integrity and ethics (AIE), which is essential for their personal development and future professional careers. This paper reports on a project which employs an augmented reality (AR) interface accessed on mobile devices to bring AIE scenarios alive for students in everyday campus contexts. Mobile learning paths called ‘Trails of Integrity and Ethics’ (TIEs) have been created on Hong Kong university campuses, with students walking through study locations where ethical dilemmas might arise, and using an AR app to learn about, consider and respond to a range of problematic scenarios. In addition, subject-specific TIEs have been developed in which students face ethical dilemmas specific to their disciplines, and are tasked with responding according to professional norms and standards.

After the first 2 years of this 4-year funded project, more than 1000 students have participated in the TIEs. Analysis of data from their mobile device clickstreams, pre- and post-trail reflective texts and user experience surveys has led to encouraging initial findings. There is some early evidence suggesting that the mobile AR trails have helped students to become more active and engaged in their learning of abstract conceptual knowledge about AIE, and that their perspectives on AIE have changed as they have begun to link ethical dilemmas on the TIEs with their everyday realities.

## Introduction

One of the most important goals of tertiary education in the twenty-first century is to instil in students the importance of behaving ethically and with integrity (Thomas and Zyl 2012). Students are expected to progress quickly and perform at a high level at university, in part due to the spread of information and communication technologies (ICTs) into all areas of higher education and indeed into most of the professions into which students will graduate. While there is no doubt that ICTs offer numerous ways of enhancing student learning and engagement (Gordon 2014; Pegrum 2014), their presence and ease of use may simultaneously facilitate unethical behaviours or low-integrity actions (Ashworth et al. 2003; Owunwanne et al. 2010; Palmer et al. 2017), especially when students are under pressure.

It was against this background that the project, *Reinforcing the Importance of Academic Integrity and Ethics* (*AIE*) *in Students through Blended Learning*—*A Deployment of Augmented Reality* (*AR*) *Applications* (*AIE*-*AR Project*), which employs mobile AR technology to support the development of students’ understandings of academic integrity and ethics, was funded by the Government of the Hong Kong Special Administrative Region through the University Grants Committee (UGC). Running from 2014 to 2018, the project is led Hong Kong Baptist University (HKBU), with three partner universities, The Chinese University of Hong Kong (CUHK), The Education University of Hong Kong (EdUHK), and The Polytechnic University of Hong Kong (PolyU).

This study addresses the research question: ‘Can the TIEs using AR technology help change students’ perspectives on AIE?’ Reporting on data obtained approximately 2 years into the project, this paper presents initial findings which go some way towards answering the question under investigation.

## A brief review of related concepts

### Academic integrity and ethics

The phrase ‘academic integrity and ethics’ is problematic as it has various interpretations and is usually employed in the context of student misconduct, such as plagiarism or falsification of data. According to the Center for Academic Integrity (CAI 1999, p. 4), academic integrity can be defined as ‘a commitment, even in the face of adversity, to five fundamental values: honesty, trust, fairness, respect, and responsibility. From these values flow principles of behaviour that enable academic communities to translate ideals into action’. Academic integrity also denotes ‘coherency between promises or rhetoric and actions’ (Gallant 2008, p. 10). For the purpose of this project, the phrase ‘academic integrity and ethics’, abbreviated as AIE, refers to the commitment of all parties to ethical principles for educational purposes and for fairness in academic settings.

Studies have suggested that premeditated plagiarism is rare (Boden and Holloway 2004); panics over deadlines and misunderstandings about how to handle information are the causes most frequently cited by students (Carroll 2003; Curtis and Popal 2011). However, a number of studies have also reported that students may intentionally engage in plagiarism when they have a perception that this will go undetected or unpunished (Hansen and Anderson 2015; Kwong et al. 2010; Palmer et al. 2017; Park 2004).

According to Scanlan (2006):

the best way to diminish academic misconduct is to prevent it. Strategies useful in preventing dishonest behaviour among students include integrity training complemented with course-level reinforcement, faculty role modelling and application of selected prevention strategies. (p. 180)

With prevention rather than detection and punishment in mind, it is opportune to deploy ICTs to help combat a phenomenon that ICTs have in part facilitated.

### The learning potential of mobile augmented reality

In a recent edited collection, Traxler and Kukulska-Hulme (2016) make it clear that they view ‘contextual mobile learning as the next generation’ of mobile learning. Kinshuk (2015) argues that, operating at the interface between new technologies and new pedagogies, educators should be exploiting ‘the potential of location-aware context-sensitive approaches that are emerging as [the] successor of [the] Web 2.0 paradigm’ (p. 1). One of the most important interfaces to have emerged to support such learning in context is AR. In a broad conceptual definition, which is of most value in education (Chow et al. 2015), AR can be seen as referring to any dynamic presentation of contextually relevant information and communication channels in a real-world setting (with a narrower, technocentric definition referring to a particular kind of visual superimposition of these digital channels on a view of the real-world setting, for example on the screen of a smart device held up to that setting) (Bacca et al. 2014; FitzGerald et al. 2012). The 2016 Higher Education *Horizon Report* suggests that AR ‘can help students learn by placing course content in rich contextual settings that more closely mirror real-world situations in which new knowledge can be applied’ (Johnson et al. 2016, p. 40).

With the advent of mobile AR, then, it has become increasingly possible to complement more formal classroom environments with a situated learning approach (Brown et al. 1989; Lave and Wenger 1991), where learners construct knowledge within the everyday social and physical contexts in which they find themselves (Comas-Quinn et al. 2009; Pegrum 2014). According to Huang et al. (2016), situated learning ‘emphasizes the importance of the “person-plus-the-surroundings” concept, where the “surroundings” include learning environments, activities, and peers’ (p. 265). Consequently, there is typically a strong emphasis on collaborative learning and co-construction of knowledge (Dunleavy and Dede 2014; Naismith et al. 2004; Radu 2014).

One of the key advantages of AR is that it can help make the invisible visible—allowing students to ‘See the Unseen’ (Dunleavy 2014, p. 32)—or can at least highlight aspects of the environment to which students might otherwise pay little heed, drawing their attention to the most relevant content (Radu 2014, p. 1540). Moreover, students can become involved in more active learning when, for example, they create their own digital annotations geotagged to real-world locations (FitzGerald et al. 2012). Going a step further in content creation, students can structure AR-based learning activities, treasure hunts or trails for their peers.

## Methodology

### Design of the trails of integrity and ethics

As the implementation of AR in education is rather new, there is at present only a limited number of published studies of its effectiveness. The current study takes an exploratory approach to ascertain the usefulness of AR in educating students on the abstract concepts of AIE. Its purpose is not to compare AR and non-AR approaches, but rather to explore what kinds of benefits AR might offer for students’ learning.

This project involved designing learning experiences for students in the form of AR learning trails, each consisting of a repository of AIE scenarios which students could access on their own mobile devices on location at various checkpoints around campus, as indicated on a map distributed at the start of the learning activity. When engaged in these TIEs, students could make use of their devices at each location to retrieve relevant information, discuss responses with peers and discover the consequences of their choices—with the option to revise those choices if necessary. By asking students to engage with AIE issues such as disrespect for intellectual property, or fabrication of research data, in on-campus locations where such temptations might arise, the aim was to bridge the gap between formal AIE classes and the application of learning in everyday settings, while motivating students to engage with content that some might perceive as dry and uninteresting, and even as irrelevant to their own studies.

Following a lengthy investigation of possible AR software, the decision was made to use the *Mobxz*/*AR*-*Learn* platform (the first version of the platform was called *Mobxz*, a generic name supplied by the vendor; with further development, the latest version is called *AR*-*Learn*, named after our project) designed specifically for the deployment of learning trails, and which had already been successfully used in Singapore (Chow et al. 2015). With built-in support for geolocation mapping, Bluetooth activation, image/object recognition and QR code scanning, the app is available for both Android and iOS devices. Learning trail contents can be simply authored using Microsoft PowerPoint and the iSpring Suite, and then converted into HTML5 files, which are in turn uploaded onto a web server dedicated to serving the TIEs.

**Fig. 1:**
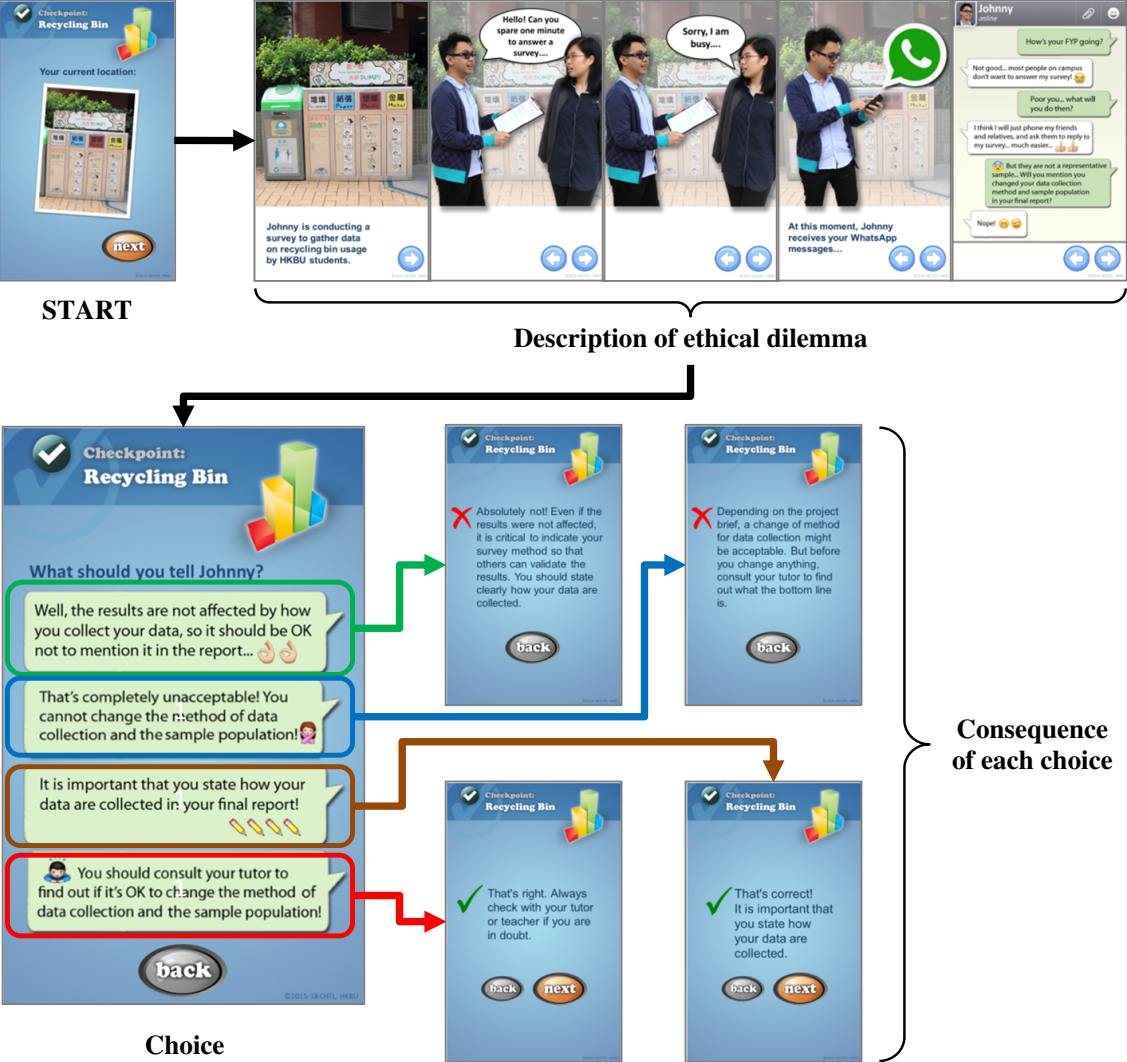
An example checkpoint (data falsification) from TIE-General

The first trail established, based on a prototype called TIE-1, was named TIE-General, and covers ethical scenarios relevant to students in all disciplines. It is a short, four-point trail set as a collaborative, student-centred activity to complement formal AIE classes. TIE-General was developed iteratively and regularly improved on the basis of data collected from the student participants. These improvements involved, amongst other things, reducing the amount of written text and including more multimodal elements, developing the characters in the scenarios presented, using more colloquial language and emphasising the competitive, gaming element. In the latest version of TIE-General, the four checkpoints involve activities relating to the following four ethical dilemmas:

i. *QR code*-*enabled plagiarism scenario in a classroom context*: Students are presented with a scenario involving a character who, with insufficient time to complete an assignment by the set deadline, considers copying from a classmate. After agreeing on which action to take in response, students receive an explanation of the likely consequences and, if necessary, guidelines on a more appropriate course of action.
ii. *GPS*-*enabled citation scenario at a statue of Sun Yat*-*Sen inscribed with a quotation*: In this scenario, students are asked to decide under what circumstances they would need to provide an attribution for this quote, and when they would not. Students receive additional relevant details about correct answers and/or feedback about erroneous ones.
iii. *Image recognition*-*enabled resources scenario triggered by students photographing a library book return box*: In this scenario, a character considers hiding a sought-after textbook on another shelf in the library so that he can consult it whenever needed. Students receive information on how other library users are likely to be affected, and are asked to collaboratively generate suggestions on how to convince this student not to commit this selfish act.
iv. *Bluetooth*-*enabled data falsification scenario triggered when students are in the vicinity of a recycling bin*: In this scenario, a character carrying out a survey on recycling considers making up for a lack of data by calling up relatives and friends to complete the survey, without reporting the change of data collection method in the final report. Students are required to consider whether this is ethical, whether the tutor should be consulted and what penalties there might be. Figure 1 shows screenshots relating to this ethical dilemma.

Originally established at HKBU, by the end of 2016 TIE-General had been transplanted to the three partner institutions and adapted as necessary to their available campus locations, with over 900 students experiencing this TIE across the four campuses. In addition, subject-specific TIEs have been developed at HKBU as follows: TIE-HT (Hall Tutors), TIE-Hum (Humanities), TIE-LabS (Laboratory Safety), and TIE-SR (Sports and Recreation). It should be highlighted that while all TIEs except for TIE-HT are in English, as this is the main medium of instruction in all four partner institutions, students’ commentaries are often bilingual; and on TIE-SR, scenarios are presented through videos in colloquial Cantonese (a dialect of Chinese dominant in Hong Kong) with English subtitles, but the questions and explanations are framed in English. This design has the added advantage of helping students, many of whom are not native speakers of English, and some of whom are attending an English-medium institution for the first time in their lives, to learn everyday English relevant to life on campus (on TIE-General) or genre-specific English relevant to their disciplines (on the subject-specific TIEs).

While the concepts and scenarios depicted in TIE-General are rather typical, the same cannot be said for the subject-specific TIEs; they have variations which may deviate a little from the AIE area as they focus on professional standards and ethical behaviour suited to specific disciplines or situations. For example, TIE-SR concentrates on sporting ethics, while TIE-HT emphasises hall tutors’ ethical decisions. When lecturers and tutors were first approached to come up with scenarios and activities relevant to their disciplines or areas of responsibility, they found it very challenging. Interestingly, the solution came from the students themselves. The Sports and Recreation lecturer was the first to solicit assistance from students in her programme, as well as members of the programme’s alumni association. Given the importance of professional, ethical behaviour in sports, both students and graduates found the conceptualisation of scenarios to be a meaningful and relevant exercise. In the end, 30 of the best student- and alumni-developed scenarios were converted into learning activities for incorporation into TIE-SR. This TIE has been experienced by 111 students in groups of 2 to 3, working on 2 different campuses. Students were asked to analyse the scenarios before providing responses, while the activities also required them to research and discuss key issues relating to sports ethics.

### Data collection and analysis

This 4-year funded project began in the fall of 2014, and by the end of December 2016, most of the planned development and deployment had been completed. In these 24 months, more than 1000 undergraduate and postgraduate students from all four partner institutions had explored issues of AIE and/or related issues on at least one of the TIEs implemented on their respective campuses. Students were encouraged by instructors of various academic courses to take part in the TIEs. Details of the participants are shown in Table 1.

**Table 1:** Student participants on the TIEs (January 2015—December 2016)

Trail	Area	Institution	Level	Students	Total
TIE-General	General	HKBU	Undergraduate (UG)	459	
		HKBU	Postgraduate (PG)	188	
		CUHK	Undergraduate	164	
		EdUHK	Undergraduate	90	
		PolyU	Undergraduate	24	925
TIE-HT	Hall Tutors	HKBU	Undergraduate	46	
TIE-Hum	Humanities	HKBU	Undergraduate	20^a^	
TIE-LabS	Laboratory Safety	HKBU	Undergraduate	111	
TIE-SL	Service Learning	HKBU	Undergraduate	46	
TIE-SR	Sports and Recreation	HKBU	Undergraduate	111	334
				Total	1259

^a^20 UG students participated in TIE-Hum, and their clickstream and text-mining data were collected, but they did not complete the user survey

Ethics clearance for data collection and analysis was obtained before the project commenced. Students who agreed to participate were informed that their data would only be used anonymously in reports and publications, and that they could withdraw at any point without prejudice. All videotaped lecturers were volunteers.

As is appropriate in an exploratory study which is seeking to gain a broad understanding of a relatively new area of educational technology deployment, a mixed methods approach has been adopted (Behar-Horenstein 2010; Johnson and Onwuegbuzie 2004).

### Clickstream data

Direct evidence of learning was sought in the clickstream data from the mobile app, which allowed us to make effective use of the mobile devices’ tracking capabilities, as suggested by de Souza e Silva and Sheller (2015). Clickstream timestamps were inserted within each scenario to record students’ activities/responses within the app. The time taken for each activity was calculated based on the differences between two timestamp logs, e.g. the time difference between timestamp 1 and timestamp 2.

### Text-mining of pre-/post-commentaries

While the pre-trail discussion primes students for the learning experience, and the post-trail discussion supports reflection on their learning, a comparison of the two also allows both lecturers and students to track changes in understandings.

John Dewey (1933) suggested that students do not learn from experiences, but rather they learn from *reflecting* on their experiences. In order to ascertain how situated learning affects students’ reflections, we collected their views on AIE or related issues before and after their experiences on the TIEs. In light of the practicalities of collecting data from the different groups of students described in Table 1, a variety of data collection approaches ranging from discussion boards in learning management systems (using mobile devices) to paper worksheets were deployed to gather the pre- and post-trail commentaries.

Students’ comments were then analysed to determine any changes in their perspectives, as evidenced in changed use of language. Two systems for text analysis were deployed in this project. The first was a proprietary system developed by one of the partners, EdUHK, in a related *Learning Analytics Platform* project (Li et al. 2015); this software is capable of mining texts in English and Chinese (including both the traditional characters used mainly in Hong Kong and Taiwan, and the simplified characters used in Mainland China), not just as separate sentences, but even when they are mixed together in any one sentence, as occurred regularly in the student data. The second was the *Carrot* freeware (http://project.carrot2.org/). As the first system, which was tailor-made for Hong Kong’s biliterate and trilingual environment, was the main deliverable of another project, it was developed almost in tandem with the TIEs in this project, on which it was then piloted. The already developed *Carrot* software provided a complementary perspective, including a data visualisation interface, in the text comparison analysis. Having two systems also granted us the advantage of checking the consistency of the results obtained from the data.

### User experience survey

An in-house-designed user experience survey was used to collect students’ perspectives on the usefulness of the TIEs for the learning of the abstract concepts of AIE. Survey data were collected via the AR-Learn app, after the students had completed a trail. The survey consists of six 5-point Likert scale (5 = strongly agree, 1 = strongly disagree) questions and an open-ended question to invite further comments on the trail experience and on the AR-Learn app. Analysis of the data collected follows below.

## Findings

While Table 1 shows the total number of students who participated in the TIEs within the reporting period of January 2015 to December 2016, the analysis and findings below focus primarily on TIE-General. With different students at different universities exploring TIE-General at different times, the heterogeneous nature of the data is necessarily a limitation of the project. However, at the mid-point of the project, combining all the data collected to date constitutes a considerable advantage due to the large sample size. Findings from other TIEs will be described to give further insight into students’ learning.

### Clickstream data

Mobile learning activities in this project are conducted in real-world environments as a form of situated learning (Pegrum 2014) to help students connect their learning with their everyday lives. This does, however, present challenges from a project monitoring perspective. In this case, two sets of data were collected as direct evidence of student learning, providing the prerequisites for learning analytics. Firstly, as students engaged in the learning activities on the TIEs, the clicks they made in the app were captured, so their choices were recorded. Secondly, the time they spent on the activities—including the time used to comprehend the challenge, to make choices and to process the consequences of each choice—was recorded. If students did not spend much time on an activity, for instance because the ‘dilemma’ presented was so obvious that there could only be one correct answer, their learning would be at a surface level (Biggs and Tang 2015) as they did not have to reflect in any detail on the exercise.

During the reporting period, 925 UG and PG students in various groups and at various times explored TIE-General on the campuses of the 4 partner institutions. Their attempts in the trail activities as captured by the clickstream mechanism are summarised in Table 2. The capturing of the clickstream data is highly dependent on the stability of the Wi-Fi provision (Chow et al. 2015; Wong et al. 2016). As the trail exercises were conducted over a period of 2 years in different locations, variations in the Wi-Fi provision were expected. To a lesser extent, capturing was also dependent on the makes and models of the mobile devices used by the students (Chan et al. 2015). While the amount of clickstream data collected is sizeable enough to allow us to perform reasonable analyses, we acknowledge that not all attempts from participating students were recorded. Based on the strength and stability of the campus Wi-Fi at HKBU, we estimate the capture rate to have been around 60% to 70% of clicks. In the ensuing discussion of the findings, the total amount of clickstream and text-mining data collected from both UG and PG trail participants will be combined for analysis and evaluation.

**Table 2:** Summary of clickstream data captured on TIE-General at HKBU (January 2015–December 2016)

Checkpoint	No. of attempts recorded (UG)	No. of successful attempts (PG)	No. of successful attempts (ALL)
Plagiarism	762	198	960^a^
Citation and common knowledge	707	193	900
Ethical use of library resources	514	217	731
Data falsification	521	183	704
Total attempts	2504	791	3295
Total no. of students	UG–737	PG–188	ALL–925

^a^Plagiarism was the first checkpoint and some students had to repeat their attempts due to unstable Wi-Fi or inexperience with the app

**Fig. 2:**
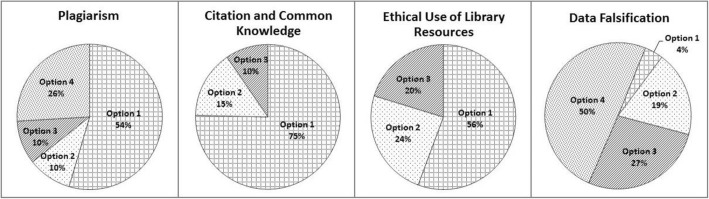
Clickstream data analysis of ethical choices (January 2015–December 2016)

The analysis of the clickstream data collected from these students for the four checkpoints on TIE-General shows that the average time spent on considering the dilemmas was reasonable (Table 3), while the choices made varied (Fig. 2). Table 3 shows that in general, students spent more time on the two scenarios, Plagiarism and Data Falsification, than on the other two scenarios, Citation and Common Knowledge, and Ethical Use of Library Resources. This may be because the former two scenarios offered three as against four choices, or because the dilemmas were genuinely more ambiguous, or a combination of both. Certainly, when varying choices were made by students, this indicates that the correct answers were not immediately apparent to them (Fig. 2).

**Table 3:** Clickstream—time on tasks (*N* = 925)

	Number of attempts	Total time spent (in min)	Average time spent (in min)
Plagiarism	960^a^	2518.55	2.62
Citation and common knowledge	900	1367.60	1.52
Ethical use of library resources	731	793.17	1.09
Data falsification	704	1330.60	1.89

^a^Plagiarism was the first checkpoint and some students had to repeat their attempts due to unstable Wi-Fi or inexperience with the app

### Text-mining based on pre-/post-reflective texts

For TIE-General, students participated in the trail under different circumstances; some trail activities were conducted during class time in credit-bearing courses, while others were organised as co-curricular activities which students joined voluntarily. In order to help students, particularly the first year undergraduates, focus their attention on AIE matters, at least one question before and one after the trail were asked to assist their reflections. The usual pre-trail question was, ‘In your own words, what is your understanding of Academic Integrity and Ethics?’, while the standard post-trail question was, ‘What have you learnt about Academic Integrity from the learning trail?’. These students were also invited to use keywords or phrases to describe their understandings, without any specification of the number of words required; for the post-trail submissions for TIE-General, students were expected to comment on each of the four tasks. TIE-General was also conducted as a teaching and learning activity in a compulsory class for PG students when academic integrity and ethics were the topics of discussion. They were asked to submit 200 words to a learning management system (LMS) discussion forum responding to the question, ‘In your own words, what is your understanding of Academic Integrity and Ethics (AIE)?’, usually at the start of the semester before the topic of AIE was even mentioned. The post-trail question, ‘What have you learnt about Academic Integrity from the learning trail?’, was the final part of a post-trail worksheet that students were required to complete in class, with a maximum word count of 200. For students who participated in the trail exercises as co-curricular activities, they could write their reflections in either Chinese or English, or both.

To perform the analysis, keywords and phrases, in both Chinese and English, were fed into the *Learning Analytics Platform*, as it allows keywords to be defined so that the students’ commentaries can be grouped accordingly. For the purpose of clarity, Chinese terms were translated into English for presentation in this paper. For example, ‘欺騙/欺詐’ were translated as ‘cheat/cheating’, which were grouped together as one concept; ‘常識/共識’ were translated as ‘common knowledge’; and ‘誠實/誠實的’ were translated as ‘honesty/honest’, etc. To maintain consistency in the presentation, the bar charts are ordered from largest to smallest according to the frequency of occurrence of concepts in the post-trail texts.

**Fig. 3:**
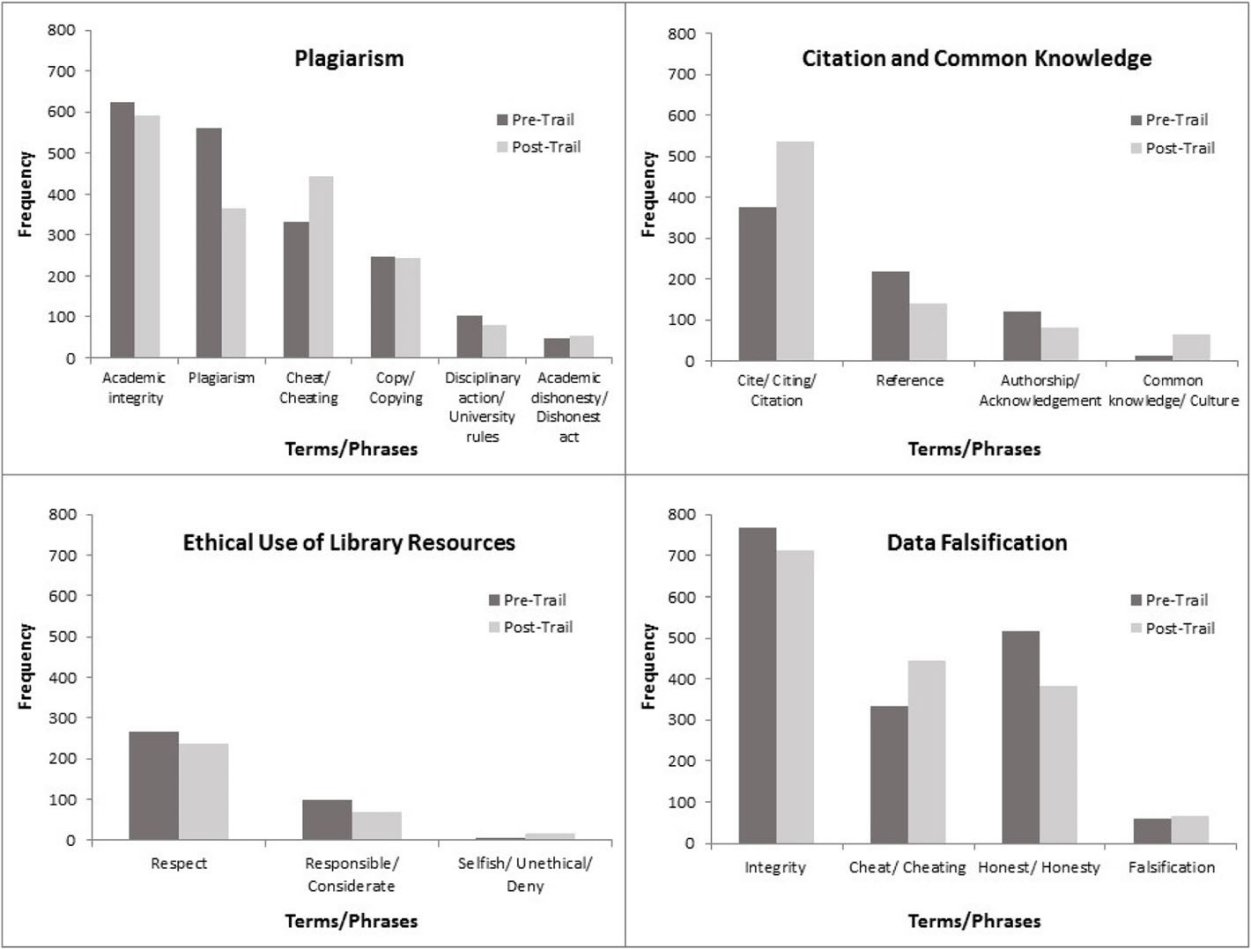
Comparison of students’ pre-/post-TIE-General reflections on the four AIE issues (January 2015—December 2016; *n* = 925)

The results of comparing pre- and post-trail reflective texts were highly encouraging. Figure 3 shows the comparison of student comments for the four AIE issues before and after their participation in TIE-General across all campuses that conducted activities on this TIE. There are decreases in the use of the terms ‘Academic Integrity’ (− 5%) and ‘Plagiarism’ (− 35%), but increases in the use of slightly more specific terms like ‘Cheat/Cheating’ (+ 33%), ‘Cite/Citing/Citation’ (+ 43%) and ‘Falsification’ (+ 8%). We also see the emergence of some interesting patterns; the increase in ‘Common Knowledge/Culture’ is significant: a jump from 13 counts to 64 (+ 392%); and the increase in the negative concept ‘Selfish/Unethical/Deny’ (+ 143%), although the numbers of occurrences are smaller, may suggest a more emotional response to the failure to behave with integrity. Some of these terms bear a direct relation to the key concepts explained in the relevant scenarios, which makes the respective increases very encouraging.

Overall, while the results are still preliminary, there may be some early indications of a shift towards the development of more specific conceptualisations of AIE, and more concrete and personalised understandings of it. To further explore this shift, we can look to the subject-specific TIEs for more insights (see below).

**Fig. 4:**
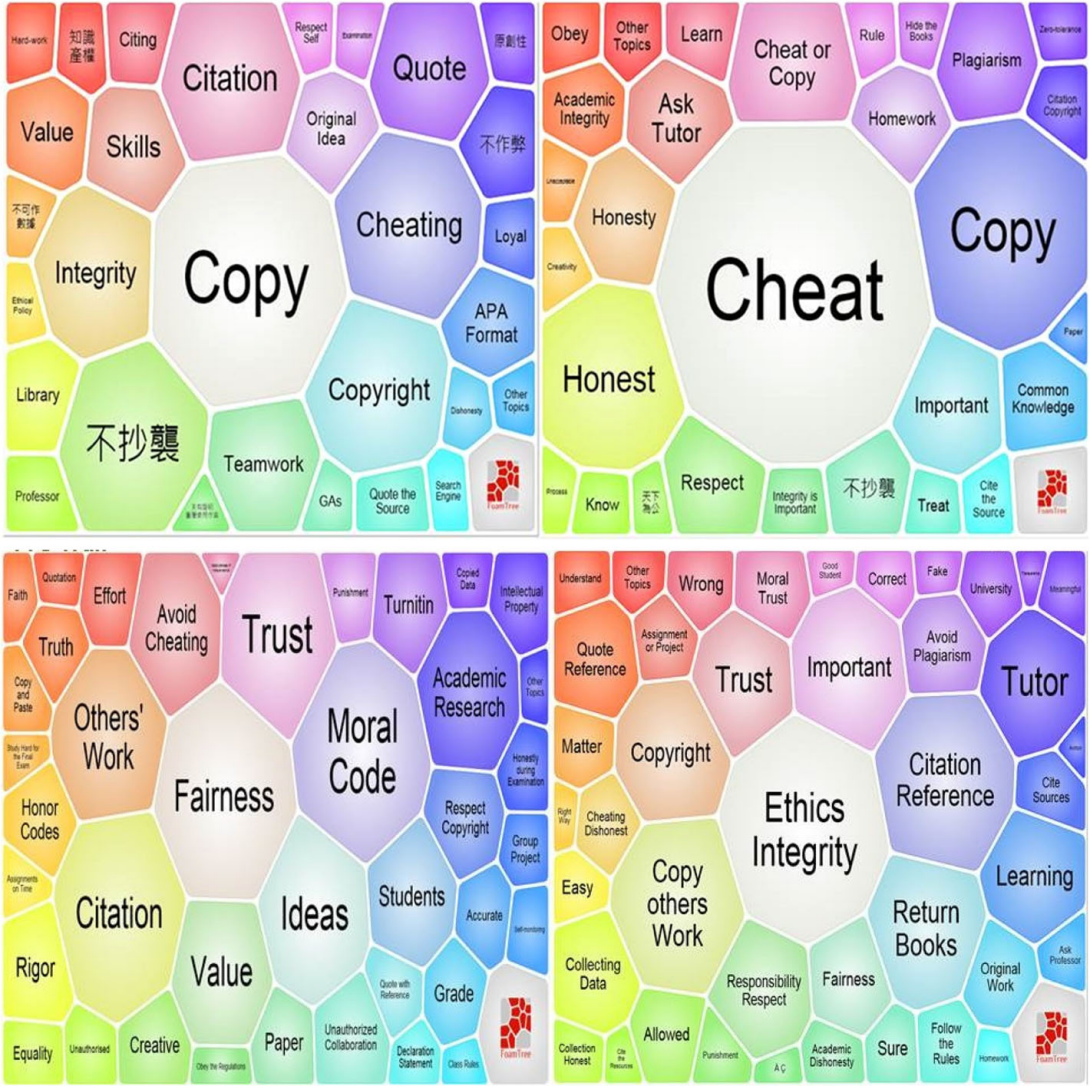
Pre- (left) and post- (right) trail text comparison (TIE-General). (Top) First year UG students at orientation (*n* = 90). (Bottom) Year 3 Business major students (*n* = 195)

Figure 4 shows a further comparison between the pre- and post-trail responses of two different groups of UG students—a group of HKBU first year undergraduates exploring TIE-General at student orientation before classes began, and a class of HKBU year 3 Business majors in a core course about business ethics—visualised through *Carrot*’*s* ‘*FoamTree*’ diagrams. *Carrot* is available freely and its operations are different from that of the EdUHK-designed learning analytics system. *Carrot*’*s* algorithm counts the frequency of terms by pattern matching, and the main parameter that can be adjusted is how finely or coarsely the matching is to be done. Hence, Chinese and English terms are considered as different patterns, and it is not possible to combine terms as was done in the learning analytics system—like putting ‘plagiarism’, ‘plagiarise’ and ‘抄襲’ together as one pattern of ‘plagiarism/plagiarise/抄襲’. Furthermore, it is not possible to control how *Carrot* combines or separates the terms in its matching. For example, the texts from students may contain the terms ‘cheat’, ‘copy’ and ‘cheat or copy’ in separate sentences. *Carrot* classifies these into three patterns: ‘cheat’, ‘copy’ and ‘cheat or copy’, and then counts the corresponding frequency for each pattern. Nevertheless, *Carrot* is useful in examining the occurrence of terms without predefined keywords, and it presents a helpful visualisation of the results. In this project, the broad similarities between the results in *Carrot* and the EdUHK-designed learning analytics system help confirm the robustness of the overall text mining approach.

In Fig. 4, comparison of the pre- and post-trail texts of the first year students shows that while the concept of ‘plagiarism’ (note: ‘不抄襲’ means ‘do not plagiarise’) occurs at a similar level in pre- and post-trail comments, the more active and concrete ‘cheat’ and ‘copy’ gain considerable salience. For the year 3 students, the abstract concept of ‘plagiarism’ is largely replaced in the post-trail commentaries by much more concrete ideas about what constitutes ethical or unethical behaviour, ranging from [ask advice from] ‘Tutor’ to [not] ‘Return Books’. Comparing Fig. 4 with Fig. 3, it is encouraging to see that, despite the differences in the pattern matching algorithms and keyword comparison controls, the terms are indeed similar (as highlighted in the tables for the term clusters in the Appendices 1 and 2), with ‘cheat’, ‘copy’, ‘honest’, ‘respect’, ‘citation’, etc., dominant in the post-trail commentaries. This reinforces the notion of students shifting from abstract concepts to more concrete and personalised understandings of AIE matters through their participation in the trails.

### A subject-specific TIE: TIE-Sports and Recreation

**Fig. 5:**
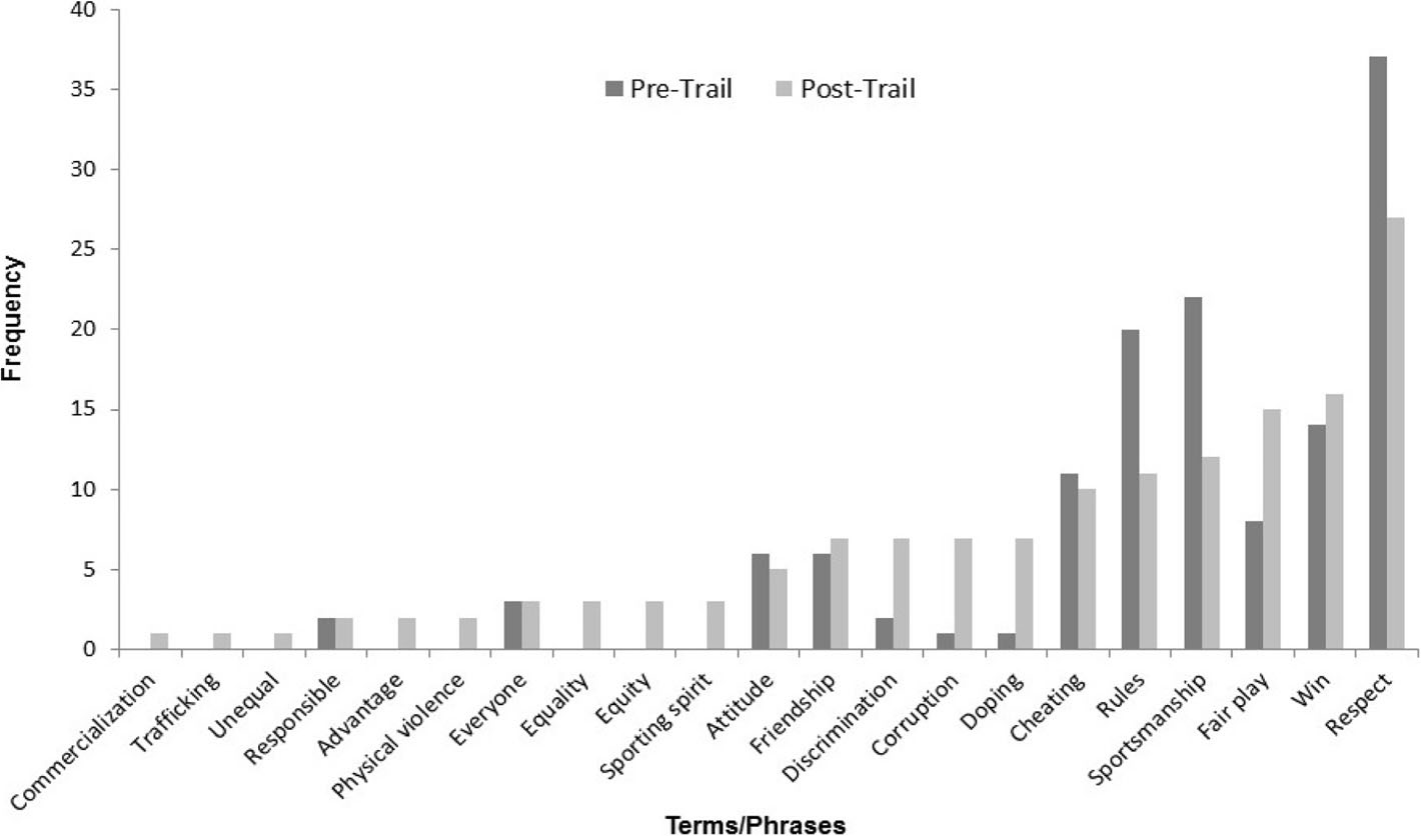
Comparison of UG students’ (*n* = 111) pre-/post-TIE-SR reflections (semester 1, 2015 and semester 1, 2016). Pre-trail question: What is your view on sports ethics? Post-trail question: From the learning trail, what have you learned about sports ethics? Note: For the purpose of clarity, some words/phrases have been shortened for presentation in Fig. 5 as follows: Excessive commercialization ➔ Commercialization, Unequal opportunities ➔ Unequal, Everyone else is doing it/Everyone else/Everybody else/Everyone/Everybody ➔ Everyone, Winning is the only thing/Winning/Win ➔ Win

TIE-SR is an exemplary trail amongst the operational TIEs, as demonstrated by the comparison of pre- and post-trail vocabulary in Fig. 5, where it can be seen that there is a considerable increase in the use of specific terminology directly relevant to sports ethics. As explained earlier, students are studying in English and need to acquire English-medium vocabulary within the discipline of sports and recreation. Regarding the results, on the one hand, the post-trail responses show an increase in the use of positive terminology like ‘fair play’ and ‘friendship’, and the emergence of terms like ‘equality’, ‘equity’ and ‘sporting spirit’. On the other hand, there is a considerable increase in the use of negative terminology like ‘discrimination’, ‘corruption’ and ‘doping’, alongside the emergence of terms like ‘physical violence’, ‘excessive commercialisation’, ‘unequal opportunities’ and ‘trafficking’, suggesting a more detailed understanding of the many ways in which ethical behaviour in sports can be undermined. While both the positive and negative concepts here are represented primarily by abstract nouns, they do suggest the development of a more specific and nuanced understanding of the positives and negatives interwoven in sporting ethics.

There are certain similarities between the changes in TIE-General and TIE-SR, though the effect of the latter seems more profound, perhaps because of the much larger number of ethical scenarios to which students are exposed, and perhaps because of the fact that all scenarios were designed by students or alumni in the discipline who were instructed to make them relevant to everyday life for a professional in the field of sports and recreation, make them engaging through the use of multimedia elements and make them genuinely ethically challenging. The findings of TIE-SR, in particular, are in accordance with the literature on situated learning, which suggests that it facilitates the learning of abstract concepts by embedding them in relevant contexts.

### User experiences

Table 4 provides a summary of the data collected from the user experience surveys from TIE-General and TIE-SR (where PE stands for Physical Education). It is apparent that students’ interest in learning about AIE was aroused by participating in the TIEs, as evidenced in their consolidated responses to the Likert-scale items in the user experience surveys. Students were asked to complete the user experience surveys at the end of the trails, as the last exercise in the AR app, or on separate survey sheets. Numerous positive responses to the open-ended question highlighted the motivational advantages of this ‘new way in learning’, and notably the gamified approach which ‘transform [ed] boring contents into vivid games’. Other responses emphasised pedagogical advantages centred on a collaborative, constructivist approach—‘[m] ore interaction than just listening to people talk’, read one typical comment—and the embeddedness of the learning which, as another comment pointed out, allowed ‘the principle [s] of academia’ to be ‘well memorised’.

**Table 4:** Consolidated Likert-scale responses to user surveys

Survey statements	All TIES	TIE-SR (*n* = 111)		
	(*n* = 1239)	Group A (PE major, campus 1)	Group B (non-PE major, campus 2)	Group C (PE major, campus 2)
1. I find this App easy to use.	3.74	4.08	3.96	3.26
2. My interaction with this App is clear and understandable.	3.91	4.02	4.00	3.23
3. This App makes learning academic integrity and ethics more interesting.	3.89	3.99	4.00	3.29
4. Working with this App is fun.	3.73	3.83	4.12	3.26
5. The WiFi connection is stable.	3.27	3.24	3.72	2.75
6. My overall usage experience with this learning trail is good.	3.80	3.85	4.16	3.27

While the motivational and pedagogical aspects of the trail were generally evaluated positively, however, it is clear that there were issues with the technological aspects, especially the stability of the Wi-Fi. This was reflected in a considerable number of references to poor Wi-Fi in the open-ended responses, with some students suggesting that greater use might be made of QR codes because of issues with Wi-Fi and image recognition on some occasions. The issues with Wi-Fi become even clearer in Table 4, comparing the experiences of three student groups on TIE-SR, with groups A and B completing this trail on a campus with superior Wi-Fi coverage, and group C completing it on a campus with limited coverage. Students in group C gave the lowest rating overall about their experience on the trail due to the poor Wi-Fi. Nonetheless, it is encouraging that despite the Wi-Fi issues for group C, overall evaluation of the learning experience remained positive. Typical comments were not dissimilar to those received regarding TIE-General: ‘it provided a new way of learning’; ‘nice interactive game’; ‘it enhanced interaction’; ‘the most memorable experience’; and ‘good for us to learn the meaning [of] ethics’.

Significantly, TIE-SR also received overwhelmingly positive feedback from those involved in its creation. In the course of group meetings between the lecturer, and students and alumni who designed scenarios, the latter indicated that they valued the learning experience involved, and that they felt a real sense of achievement as they overcame conceptual and technological challenges in creating scenarios for trail participants.

Lecturers in this project were not surveyed but rather interviewed, with the recordings made available on the project website. The project seems to have had a significant impact on the lecturers as well. Interviews revealed that due to this project, educators had conducted significant research on issues of professional integrity and ethical standards in academia as well as within their specific disciplines. They claimed to have a better understanding and improved awareness of AIE issues, which in turn helped them to better assist their students in the learning of such abstract concepts. Participating educators found the AR app innovative, and considered that involving students in mobile learning in a highly structured manner led to more active engagement in the learning. Several subject lecturers are now considering deploying AR for their discipline contents, not just for the AIE issues focused on in this project.

## Discussion and conclusion

Reporting on the outcomes of the first 2 years of a 4-year, Hong Kong-government-funded mobile AR research project, in which TIEs have been created and trialled with well over 1000 student participants, this paper has attempted to answer the research question: ‘Can the TIEs using AR technology help change students’ perspectives on AIE?’. Drawing together quantitative and qualitative data obtained from mobile app clickstreams, text mining of pre- and post-trail commentary and user experience surveys, it has provided preliminary evidence of the learning benefits of the use of cutting-edge AR technology to facilitate students’ understandings of the abstract concepts of AIE.

First, it was found that students were very engaged in the contextualised learning facilitated by mobile AR, as reflected both in their responses to Likert-scale items and open question on the user experience surveys. While there was a general appreciation of the novelty of this kind of learning approach, it was notable that a number of students commented specifically on the gamification aspects of TIE-General and TIE-SR. This is in line with the research literature on mobile AR, which suggests that there are motivational gains (Radu 2014), particularly where gamified elements are introduced (Schmitz et al. 2012; Bacca et al. 2014).

Second, one likely reason for students’ increased motivation was the linkage between abstract concepts and students’ everyday lived realities, as reflected in the changed vocabulary they used in their post-trail commentaries. It was encouraging to see an increase in the use of more concrete terms, suggesting that students had more specific understandings of abstract concepts like ‘academic integrity’ or ‘ethics’. In some cases, there were shifts towards the use of more emotive terminology, suggesting a personal connection with the learning taking place, and towards the use of negative terminology, suggesting a growing appreciation of the negative consequences of failing to act with integrity. TIE-SR, in particular, showed the emergence of students’ higher awareness of the importance of behaving ethically in sports with both positive and negative comments. In essence, it would seem that some changes in students’ perspectives on AIE took place after they had explored TIE-General and TIE-SR. This is in line with the research literature on situated learning, which stresses the links students make between the classroom and the real-world environments where their learning applies, as they collaboratively construct understandings within their physical and social contexts (Comas-Quinn et al. 2009; Huang et al. 2016).

There are a number of limitations in the current study. This paper reports on only the first 2 years of the 4-year project, during which the trails were gradually developed and iteratively improved, with appropriate data collection mechanisms being implemented as the nature of the trail design and learning became clearer to the researchers. To date, the motivational and pedagogical benefits of the trails have been partially compromised by technological issues, notably the sometimes limited Wi-Fi coverage, and the iterative development of the trails meant that some small changes in setup and, consequently, data collection occurred. As mentioned earlier, despite the obvious advantages of analysing data from a large sample, there are potential issues with combining heterogeneous data together for analysis; this will be addressed in future studies where larger data sets will be available for individual trails at individual institutions. In general, there is scope to extend the project in terms of both the length of time and the number of participants, which will be especially important in seeking to more clearly identify shifts in students’ language use between their pre-trail and post-trail commentaries. Moreover, while the early results are encouraging, changes in students’ perspectives do not necessarily lead to changes in their behaviours, a point which could be addressed in a future study.

As an exploratory study, the current research did not involve a control group for comparison, though it did involve both pre- and post-trail qualitative data being collected from participants and analysed for changes, as described in previous sections. There is some debate on the issues around control groups (Boruch 1975; Conner 1980), and there are questions about student equity if some groups are not exposed to potentially useful new technological approaches. In any case, this study did not set out to compare AR with non-AR pedagogies; nevertheless, the lack of a control group may be perceived as a limitation and, if deemed appropriate, it is an area that could be addressed in other future studies.

In response to a quandary regarding how to create sufficient numbers of trail scenarios, how to ensure these were authentic and how to express them in language to which students could relate, one lecturer invited current students as well as alumni to design scenarios for TIE-SR, and eventually went on to select the best of these for use in the final trail. Subsequently, other TIEs have adopted this approach of deploying students to assist in scenario and activity design. As a result, this kind of student input has become the default for new trails. From a staff perspective, this has enhanced the ability to conceive of and deliver trails. It is still too early to draw any conclusions about this from a student perspective. Nonetheless, students and alumni involved in the design process reported benefits for their own learning as they conceptualised and constructed learning experiences for their more junior peers. Further exploration can be conducted in line with social constructivist learning principles, where students are able to teach and learn from each other (Pegrum 2014). This links to the newest strategies associated with the implementation of mobile AR platforms, where students are asked to design learning experiences for other students (LDR 2016; Rockmoon 2012), so that they are no longer passive consumers but rather become active creators of learning experiences for themselves and their peers.

In the remaining 2 years of the project and beyond, the initial TIE-General, TIE-SR and other TIEs (see Table 1) will be used by larger numbers of students, while more TIEs, currently under development, will also be implemented for the first time. Transplantation of TIEs amongst partner institutions, and the adoption and adaptation of each other’s AIE scenarios, will continue. To address some of the aforementioned limitations, there will be a fixed and systematised data collection system in place from August 2017, which will provide a new, larger dataset to ascertain whether there is continued evidence of the emerging trends this paper has outlined. It is anticipated that TIE-General will become a standard induction activity for new students, with relevant subject-specific TIEs being explored by students as they go on to specialise in their studies. Thus, students’ immersion in the learning of abstract AIE concepts will be more comprehensive and iterative. As using the subject-specific TIEs amounts to a learning innovation, an alternative to setting up control groups in future studies could involve comparing the learning of students exposed to subject-specific AIE issues through exploring subject-specific TIEs, with that of students who have learned about similar issues by other means. Further exploration will be conducted in how best to integrate additional gamification elements, and how to offer students more options for generative learning—that is, creating their own trails or elements of trails—so as to reinforce their own and their peers’ learning.

It is hoped that in this way, the TIEs, with their apparent scope for fostering engaging learning, personalised learning and generative learning, will contribute even more substantially to students’ understandings of AIE and, in particular, their readiness to apply those understandings in the everyday contexts where they are required. If the ongoing data collection and analysis continue to indicate positive outcomes, it is anticipated that in time similar trails might be transplanted to, or set up in, other tertiary institutions, and might perhaps be implemented even more broadly within the education sector.

## Appendix 1

**Table 5:** First year students’ pre-/post-trail terms/phrases clusters from Carrot

Pre-Trail		Post-Trail	
Copy (22)*	Citing (3)*	Cheat (43)*	Know (3)
Cheating (12)*	Dishonesty (2)*	Copy (19)*	Learn (3)
不抄襲 (12)*	Ethical policy (2)*	Honest (13)*	Cite the source (2)*
Copyright (11)*	Examination (2)	Cheat or copy (9)*	Creativity (2)
Integrity (9)*	GAs (2)	Important (7)	Hide the books (2)
Quote (8)*	Hard-work (2)	Honesty (6)*	Integrity is important (2)*
Teamwork (7)	Loyal (2)	Plagiarism (6)*	Obey (2)
Skills (5)	Professor (2)	Respect (6)*	Paper (2)
APA format (4)	Respect self (2)*	Ask Tutor (5)	Process (2)
Library (4)	Search engine (2)	Common knowledge (5)*	Rule (2)*
Original Idea (4)*	不可作數據 (2)*	Homework (4)	Treat (2)
Quote the source (4)*	原創性 (2)*	不抄襲 (4)*	Unacceptance (2)*
Value (4)*	未有聲明重複使用作業 (2)*	Academic integrity (3)*	Zero-tolerance (2)*
不作弊 (4)*	知識產權 (2)*	Citation copyright (3)*	天下為公 (2)

Numbers in brackets show the frequency/count in Carrot (*terms/phrases also shown in Fig. 3)

## Appendix 2

**Table 6:** Year 3 business ethics students’ pre-/post-trail terms/phrases clusters from Carrot

Pre-trail		Post-trail	
Fairness (27)*	Accurate (5)	Ethics integrity (29)*	Cite sources (4)*
Moral code (26)*	Equality (5)	Citation reference (20)*	Collection honest (4)*
Citation (25)*	Punishment (5)*	Copy others (’) work (19)*	Correct (4)
Trust (21)*	Quote with reference (5)*	Important (15)	Follow the rules (4)*
Ideas (18)	Copy and paste (4)*	Return books (14)	Matter (4)
Others’ work (18)	Honest(l) y during examination (4)*	Copyright (13)	Meaningful (4)
Academic research (16)	Obey the regulations (4)*	Tutor (12)	Punishment (4)*
Value (15)	Quotation (4)*	Responsibility respect (11)*	Wrong (4)
Avoid cheating (14)*	Self-monitoring (4)	Trust (11)*	Ask Professor (3)
Students (12)	Assignments on time (3)	Learning (10)	Easy (3)
Rigour (9)	Copied data (3)*	Collecting data (9)*	Fake (3)
Turnitin (9)	Declaration statement (3)*	Avoid plagiarism (8)*	Sure (3)
Unauthorised collaboration (9)	Faith (3)	Fairness (8)*	Understand (3)
Respect copyright (8)*	Group project (3)	Quote reference (8)*	Action (2)
Creative (7)	Unauthorised (3)	Allowed (6)	Cite the resources (2)*
Effort (6)	Absoluteness of independence (2)	Moral trust (6)*	Good student (2)
Grade (6)	Class rules (2)*	Original Work (6)*	Homework (2)
Honour codes (6)*	Study hard for the final exam (2)	Cheating dishonesty (5)*	Right way (2)
Intellectual property (6)*		University (5)	Transparen(t) (2)
Paper (6)		Academic dishonesty (4)*	
Truth (6)*		Assignment or project (4)	

Numbers in brackets show the frequency/count in Carrot (*terms/phrases also shown in Fig. 3)

## Abbreviations

AIE: Academic integrity and ethics
AR: Augmented reality
ICTs: Information and communication technologies
LMSs: Learning management system
PG: Postgraduate
TIE-HT: Trails of Integrity and Ethics-Hall Tutors
TIE-Hum: Trails of Integrity and Ethics-Humanities
TIE-LabS: Trails of Integrity and Ethics-Laboratory Safety
TIEs: Trails of Integrity and Ethics
TIE-SL: Trails of Integrity and Ethics-Service Learning
TIE-SR: Trails of Integrity and Ethics-Sports and Recreation
UG: Undergraduate
UGC: University Grants Committee
